# The promotional effect of prosocial motivation on time‐based prospective memory

**DOI:** 10.1002/pchj.738

**Published:** 2024-03-07

**Authors:** Yunfei Guo, Jiaqun Gan, Zhen Wang, Yongxin Li

**Affiliations:** ^1^ Faculty of Education Henan University Kaifeng China

**Keywords:** attention, prosocial motivation, time‐based prospective memory, viewing time

## Abstract

Time‐based prospective memory (TBPM) is the ability to remember to do a planned task at the right time. In social interactions, people are often motivated to do things for others, which reflects an important factor that influences prospective memory, namely prosocial motivation. According to the motivational cognitive model, prosocial motivation promotes TBPM by paying more attention or adopting more effective strategies. This study explored the effect of prosocial motivation on TBPM under different time‐monitoring conditions within the motivational cognitive model framework. One hundred and thirty‐one university students participated in this experiment that adopted a 2 (groups: control, prosocial motivation) × 2 (viewing time conditions: limited, unlimited) between‐subjects design. The results revealed that the prosocial motivation group had better TBPM performance than the control group under both limited and unlimited viewing time conditions. At the same time, compared with the control group, the prosocial motivation group consumed more internal attention and utilized more strategies under both viewing time conditions, and their external attention was more effective. In addition, the external attention of the prosocial motivation group was higher only when time‐monitoring was unlimited. The results of this study further extend knowledge of the motivational cognitive model and expand its scope of application, which has theoretical significance.

## INTRODUCTION

Prospective memory (PM) refers to remembering to do the planned thing in the future situation or time (Rummel & McDaniel, 2019). Some PM tasks have explicit external cues, such as remembering to hand in a document when you pass by the administrative office, which is event‐based prospective memory. There are also PM tasks that have no explicit external cues and need to be executed at a given time, for instance, remembering to pick someone up at the airport at 12 noon today, which is time‐based prospective memory (TBPM). Most laboratory PM studies have usually adopted the dual‐task paradigm, where PM tasks are inserted into an ongoing task (Hockey & Cutmore, 2021). The ongoing tasks, as basic tasks, are usually prioritized, while PM tasks may be easily regarded as secondary processing objects (Guo et al., 2023). Prosocial motivation is a psychological tendency whereby people act in ways that aim to benefit others (Lockwood et al., 2021). Although the behavior induced by prosocial motivation usually requires a sacrifice from individuals, it improves others' survival opportunities and the overall group's adaptability (Piatak & Holt, 2020). In daily life, however, we tend to plan future actions under certain motivations, and especially in social interactions, we often plan to help others to do things. PM tasks in such social situations are often considered more important and are easily prioritized (Zuber & Kliegel, 2020). Therefore, the processing mechanism underpinning PM tasks under socially motivated conditions should be different from those of general PM tasks.

The motivational cognitive model holds that prospective memory goals associated with motivation are evaluated as more important. On the one hand, this importance will prompt individuals to assign more attention to prospective memory tasks so that prospective memory tasks can be more fully processed. On the other hand, motivation can also prompt individuals to adopt more strategies, so that prospective memory tasks are in a state of spontaneous extraction (Penningroth & Scott, 2013a, 2014). In fact, the adoption of strategies also requires individuals to plan ahead, which requires individuals to put in more effort before the implementation of the task. So the adoption of strategies also requires individuals to put in more effort in advance. Some studies have also found that the prospective memory performance improved significantly when participants received higher importance ratings (Penningroth & Scott, 2013b; Penningroth et al., 2011) and when more attention resources were assigned to the prospective memory task (Penningroth & Scott, 2014). In short, the core view of the motivational cognitive model is that individuals will make more effort to fully process prospective memory tasks under motivational conditions. Prosocial motivation belongs to a type of motivation (Brandimonte et al., 2010). This study primarily explores the promotional effect of prosocial motivation on TBPM within the framework of the motivational cognitive model.

A few studies have explored the effect of prosocial motivation on event‐based prospective memory. For example, Brandimonte et al. (2010) told participants that the study was for the experimenter's graduation thesis to socially motivate them. The results showed that prosocial motivation improved the individual's event‐based prospective memory score, and their consumption of cognitive resources of event‐based prospective memory tasks was significantly reduced under the prosocial motivation condition. Walter and Meier (2017) further directed prosocial motivation to event‐based prospective memory tasks only. They informed participants that it was important for them to complete event‐based prospective memory tasks and found that prosocial motivation improved individuals' event‐based prospective memory performance, and the promotion of prosocial motivation did not require additional attention consumption. In conclusion, the promotional effect of prosocial motivation on event‐based prospective memory should mainly rely on the first approach within the motivational cognitive model.

The processing mechanism of TBPM is different from that of event‐based prospective memory. Event‐based prospective memory has clear external cues, and an individual's event‐based prospective memory performance can be easily improved by using strategies that do not require additional attention consumption. Examples include implementation intention (Henry, 2021) and imagination (Cottini et al., 2021). However, the key to successful implementation of TBPM lies in time estimation (Gan & Guo, 2019), and time information processing in TBPM consumes much self‐initiated attention resources (McBride & Flaherty, 2020). The TBPM tasks depend on both internal efforts and attention to external time information, which we refer to as internal attention and external attention, respectively (Guo et al., 2022). The ongoing task performance usually reflects internal attention, and the performance of viewing time usually reflects external attention (Hu & Qi, 2018). When performing TBPM tasks, people are inclined to pay more attention rather than use external strategies (Reese‐Melancon et al., 2019). Compared with internal attention, it is usually more cost‐effective to improve TBPM performance by improving external attention (Guo et al., 2022). For example, it is usually more accurate to check your watch several times to confirm whether it is 12 midday than to guess the time. Since prosocial motivation does not limit the way individuals improve their TBPM performance, they are more likely to improve their attention (especially external attention) rather than use strategies when they realize that TBPM tasks are more important. Therefore, prosocial motivation is more likely to promote TBPM through the second approach. However, the motivational cognitive model only proposes some possible ways for prosocial motivation to promote TBPM, and the actual mechanism has not been fully identified. In the following paragraph, we discuss the motivational cognitive model and the aims of this study by analyzing previous studies in detail.

Several studies have explored the impact of prosocial motivation on TBPM. For example, Altgassen et al. (2019) told children with autism in a prosocial motivation group that they were exploring the number of ongoing tasks performed by participants in 1 min and need help from the participants to press a key at 1 min. They found that the prosocial motivation group had no obvious advantage in TBPM performance, indicating that prosocial motivation did not significantly improve the children's TBPM scores. In another study, Altgassen et al. (2010) explored the TBPM performance of both older and younger people under a prosocial motivation condition. In the prosocial condition, they told participants that they would do experimenter a favor by pressing a key at 2 min while completing the ongoing task. They found that the older people showed advantages of prosocial motivation group in TBPM. However, existing studies have some limitations in manipulating prosocial motivation. For example, participants in the prosocial motivation condition were told to help the experimentor to explore how many times the participants could perform the ongoing tasks within the specified time in the two studies, so that prosocial motivation was directed to ongoing tasks not to TBPM tasks. The existing studies are unable to fully test the motivational cognitive model. Hence, the first aim of this study was to investigate the influence of prosocial motivation on TBPM and its processing mechanism. For this purpose, consistent with Walter and Meier (2017), we examined how prosocial motivation affected TBPM tasks. To understand the mechanism by which prosocial motivation influences TBPM, we asked participants to report the strategies they adopted when performing TBPM tasks in addition to assessing internal and external attention during ongoing task performance and viewing times.

This study investigate the effect of prosocial motivation on TBPM under different time monitoring conditions. It is well known that feedback can be obtained more effectively by checking the time more frequently, thus improving TBPM performance (Mioni et al., 2020). However, the opportunity to check the time is not always available in real life. When viewing time was limited, participants in the prosocial motivation condition were unable to improve their TBPM performance by consuming more external attention. Therefore, under the restricted monitoring condition, the promotional effect of prosocial motivation on TBPM may decrease. We hypothesized that the prosocial motivation group would have better TBPM accuracy than the control group. However, the processing mechanism underpinning TBPM promoted by prosocial motivation under the two conditions would be different. When viewing time was unlimited, the prosocial motivation group had higher viewing time frequency. By comparison, when viewing time was limited, the prosocial motivation group had worse ongoing task performance.

The motivational cognitive model suggests that the importance evaluation induced by motivation will encourage individuals to adopt more strategies to ensure the successful execution of prospective memory tasks (Penningroth et al., 2011). Therefore, we hypothesized that the prosocial motivation group would adopt more strategies than the control group. This study has theoretical significance. In the first instance, we preliminarily tested the boundary conditions of the promotional effect of prosocial motivation on TBPM. In addition, we investigated the processing mechanism(s) by which prosocial motivation promotes TBPM within the framework of the motivational cognitive model, and tested the applicability of the motivational cognitive model to TBPM tasks.

## METHOD

### Participants

One hundred and fifty undergraduate students, aged between 18 and 22 years, were recruited on campus. All participants were required to have no color blindness, be right‐handed, and have been healthy recently. They were required to be learning English as their foreign language, could not be majoring in psychology and could not have participated in any memory‐related experiments in the past 3 months. Participants were randomly assigned to one of four groups: control group with limited viewing time, control group with unlimited viewing time, prosocial motivation group with limited viewing time, and prosocial motivation group with unlimited viewing time. Referring to a previous study, we excluded participants whose ongoing task accuracy was less than 0.7 and who had no prospective memory response throughout the experimental procedure (Guo et al., 2023). In the end, valid data from 131 participants were retained. Among them, 34 participants were allocated to the control group with limited viewing time (*M*_age_ = 19.79 years, *N*_male_ = 12), 33 participants were in the control group with unlimited viewing time (*M*_age_ = 19.67 years, *N*_male_ = 11), 33 participants were in the prosocial motivation group with limited viewing time (*M*_age_ = 19.88 years, *N*_male_ = 13), and 31 participants were in the prosocial motivation group with unlimited viewing time (*M*_age_ = 20.06 years, *N*_male_ = 12). Each participant needed to sign an informed consent form before commencing the study. They conducted the experimental program independently and received about four dollars after carefully completing the experiment. The studies were reviewed and approved by Institutional Review Board of Henan Provincial Key Laboratory of Psychology and Behavior.

### Design

We adopted a between‐subjects design of 2 (groups: control, prosocial motivation) × 2 (view time conditions: limited, unlimited).

### Procedure

At first, participants needed to understand the instructions of ongoing tasks in detail, and we informed them that the 1‐back task was the ongoing task. Then they practiced 20 1‐back tasks. Each ongoing task was first presented with a “+” fixation point of 250 ms, followed by an English capital letter. Participants were asked to judge whether this letter was the same as the letter preceding it (the first letter had no comparison object, so there was no need to respond). If they were different, participants pressed the ‘F’ key, otherwise they pressed the ‘J’ key. The letters disappeared immediately after the participant reacted. If they did not respond, the letters disappeared after 1500 ms. A blank screen then appeared for 250 ms. If the participant's task accuracy was higher than 0.7 in the practice stage, they were allowed to commence the formal experiment; otherwise they were asked to practice again. Then, participants were required to perform another time task (TBPM task) along with the 1‐back task. When performing this task, participants needed to press the ‘0’ key for exactly 1 min. Participants in the unlimited viewing time condition could check the time by pressing the space bar at any time, and a time reminder lasting 1 s appeared on the screen, while participants in the limited viewing time condition could check the time only once during each TBPM task. Compared with the control group, the prosocial motivation group was additionally informed that the TBPM task was the focus of the experimenter's graduation thesis. If they could complete this task well, the experimenter could successfully graduate. Such prosocial motivation guidance has been proven to be effective in inducing prosocial motivation (Brandimonte et al., 2010). The experiment under all conditions comprised four TBPM tasks, and there was a total of 132 ongoing tasks and three breaks. All participants were required to rest every 68 s and the timing of each TBPM task began at 0:00. After completing the experiment, participants were asked to report whether they used any strategies to complete the TBPM task and to describe the strategy used.

## RESULTS

### Accuracy of TBPM task

**FIGURE 1:**
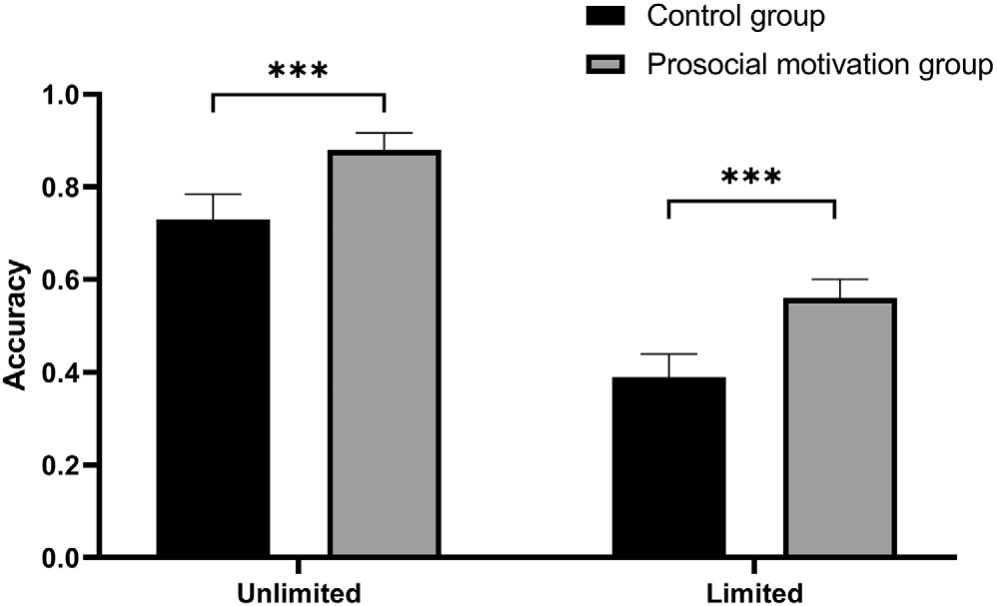
The time‐based prospective memory performance of the control group and the prosocial motivation group in different viewing time conditions. ***p < .001.

If a participant hit 0 within 5 s of 1 min, we assumed they performed the TBPM task correctly. The results of an analysis of variance (ANOVA) showed that the main effect of viewing time condition was significant, *F*(1,127) = 57.30, *p* < .001, η_p_^2^ = .31, and TBPM accuracy was lower in the limited viewing time condition than in the unlimited viewing time condition. The main effect of group was also significant, *F*(1,127) = 12.63, *p* < .001, η_p_^2^ = .09, and the TBPM accuracy of the prosocial motivation group was higher than that of the control group. By comparison, the interaction between the two was not significant, *p* > .05 (see Table 1 and Figure 1).

**TABLE 1:** The TBPM and ongoing task performance.

Groups	Viewing time conditions	TBPM task	Ongoing task	
		ACC	ACC	RT (ms)
Control group	Unlimited	0.73 (0.30)	0.88 (0.05)	629 (76)
	Limited	0.39 (0.27)	0.86 (0.03)	647 (104)
Prosocial motivation group	Unlimited	0.88 (0.20)	0.88 (0.03)	688 (88)
	Limited	0.56 (0.22)	0.85 (0.04)	716 (110)

Abbreviations: ACC, accuracy; Limited, limited viewing time condition; RT, reaction time; TBPM, time‐based prospective memory; Unlimited, unlimited viewing time condition.

### Accuracy of ongoing task

The results of an ANOVA showed that the main effect of viewing time condition was significant, *F*(1,127) = 9.28, *p* < .01, η_p_^2^ = .07, and the accuracy of the ongoing task in the limited viewing time condition was lower than in the unlimited viewing time condition. However, neither the main effect of group nor the interaction between group and viewing time condition was significant, *p*s > .05 (see Table 1).

### Reaction times of ongoing tasks

The results of an ANOVA showed that the main effect of group was significant, *F*(1,127) = 14.61, *p* < .001, η_p_^2^ = .10, and the prosocial motivation group's reaction time was slower than that of the control group. However, neither the main effect of viewing time condition nor the interaction between group and viewing time condition was significant, *p*s > .05 (see Table 1).

### Viewing time frequency

The results of an ANOVA showed that the main effects of both viewing time condition and group were significant, *F*(1,127) = 131.94, *p* < .001, η_p_^2^ = .51, *F*(1,127) = 5.43, *p* < .05, η_p_^2^ = .04, and the interaction between the two groups was also significant, *F*(1,127) = 4.74, *p* < .05, η_p_^2^ = .04. Simple effects analysis showed that the viewing time frequency of the prosocial motivation group was higher than that of the control group's only when viewing time was not limited, *p* < .05 (see Table 2).

**TABLE 2:** The viewing time performance and the number of strategies.

Groups	Viewing time conditions	Frequency	Time difference (s)	Number of strategies	Effectiveness of strategies
Control	Unlimited	2.90 (1.68)	13.93 (4.74)	0.58 (0.67)	2.47 (0.72)
	Limited	0.86 (0.17)	16.81 (3.14)	0.55 (0.56)	2.53 (0.62)
Prosocial	Unlimited	3.89 (1.89)	11.13 (5.31)	0.82 (0.68)	2.33 (0.64)
	Limited	0.89 (0.17)	14.86 (3.20)	0.82 (0.76)	2.45 (0.62)

Abbreviations: Control, control group; Frequency, viewing time frequency; Limited, limited viewing time condition; Prosocial, prosocial motivation group; Time difference, time difference of viewing time; Unlimited, unlimited viewing time condition.

### Time difference of viewing time

To further analyze the impact of viewing time, we analyzed the time difference, which was the difference between the average time‐point when a participant checked the time and 1 min, that is, the time difference between a participant's target time and the checking time. The results of an ANOVA showed that the main effect of viewing time condition was significant, *F*(1,127) = 20.34, *p* < .001, η_p_^2^ = .14, and the time difference in the limited viewing time condition was greater than in the unlimited viewing time condition. The main effect of group was also significant, *F*(1,127) = 10.54, *p* < .001, η_p_^2^ = .08, and the time difference of the control group was greater than that of the prosocial motivation group's. By comparison, the interaction between the two was not significant, *p* > .05 (see Table 2).

### Number of strategies

The results of an ANOVA showed that the main effect of group was significant, *F*(1,127) = 4.80, *p* < .05, η_p_^2^ = .04, and the prosocial motivation group adopted more strategies than the control group. However, neither the main effect of the viewing time condition nor the interaction between group and viewing time condition was significant, *p*s > .05 (see Table 2).

### Effectiveness of strategies

Examples of strategies of high effectiveness: (a) Strategic viewing time (First estimate the time, then check the time for feedback, and then repeat these two steps until participants are confident in making a prospective memory response at the target time point), (b) Counting. Examples of strategies of medium effectiveness: (a) Counting the number of ongoing tasks. Examples of strategies of low effectiveness: (a) Based on their own level of fatigue.

Two raters independently rated the strategies adopted by the participants. The raters could rate the strategies using three levels: high effectiveness, moderate effectiveness, and low effectiveness. Each level was assigned 3 points, 2 points, and 1 point, respectively. Only when two raters gave the same evaluation to the same strategy did we accept the evaluation of its effectiveness. If two raters had different evaluations, their differing opinions were resolved by discussion (Penningroth & Scott, 2013b). The interrater agreement between two raters was 86%. In the end, 51% of the strategies were identified as highly effective (such as counting, strategic viewing time, etc.), 39% of the strategies were identified as moderately effective (such as counting the number of ongoing tasks, etc.), and 10% of the strategies were identified as low effective (such as estimating time based on their own fatigue level, etc.). The results of an ANOVA showed that the main effects of group and viewing time condition were not significant, *p*s > .05, and the interaction effect between group and viewing time condition was also not significant, *p* > .05.

## DISCUSSION

This study investigated the promotion of prosocial motivation to TBPM under different time‐monitoring conditions. We found that the prosocial motivation group had better TBPM performance than the control group under both limited and unlimited viewing time conditions, indicating that prosocial motivation can improve TBPM performance. These findings were consistent with the hypothesis of this study. However, Altgassen et al. (2010) did not find prosocial motivation promoted TBPM in young people. This may have been because they directed prosocial motivation towards the ongoing task not to TBPM, so the promotion effect on TBPM could not be observed. To investigate the promotional effect specifically, we focused solely on prosocial motivation and effect on targeting task (TBPM) and the results indicated that prosocial motivation improved TBPM performance in young people (the participants in the current study). In addition, the present study also focused on the effect of prosocial motivation on TBPM for the first time under the condition of limited viewing time, and the promotional effects of prosocial motivation were found, which was also consistent with our hypothesis. In conclusion, we found that prosocial motivation can promote TBPM, which was not affected by time‐monitoring conditions.

The second aim was to test the mechanism by which prosocial motivation promotes TBPM processing within the motivational cognitive model framework. In terms of internal attention, the reaction speed of the prosocial motivation group in the ongoing task was slower under both viewing time conditions, which indicated that the prosocial motivation group slowed down their speed when executing the task. Different from the decrease in the accuracy of an ongoing task, slowing down the reaction speed of an ongoing task is a strategy, indicating that individuals deliberately reserved more attention resources for TBPM tasks (Heathcote et al., 2015). Sufficient attention is conducive to more accurate time estimation (Taatgen et al., 2007), and accurate time estimation plays an important role in the correct execution of TBPM tasks (Gan & Guo, 2019).

In terms of external attention, this study found that the viewing time frequency of the prosocial motivation group was higher only when viewing time was unlimited. However, prosocial motivation promoted TBPM under the two time‐monitoring conditions, and the changes of the two were inconsistent. Thus, although frequent time‐checking can cost‐effectively improve TBPM performance (Mioni et al., 2020), it is not the only strategy that can promote prosocial motivation. When external attention is limited, individuals can still obtain better TBPM performance by relying on other methods. We further found that the prosocial motivation group had a smaller time difference than the control group under the two viewing time conditions, which indicated that the viewing time‐point of the prosocial motivation group was closer to the target time‐point. The closer individuals get to the target time‐point to view time, the more effective time information feedback they receive from external sources, which also plays an important role in improving TBPM performance (Guo et al., 2022). Time difference is closely related to an individual's internal attention. At first, individuals estimate time conservatively, and then check to confirm how much time is left from the target (Einstein et al., 1995). More internal attention will improve the accuracy of an individual's time estimation when processing TBPM tasks (Gan & Guo, 2019; Taatgen et al., 2007). This study also revealed that the prosocial motivation group spent more internal attention with less time difference, indirectly verifying that the increased consumption of internal attention by the prosocial motivation group also improved the effectiveness of their external attention.

Of course, more effective external attention of prosocial motivation was also conducive to their estimation of the residual time interval. In particular, when viewing time was limited, only one viewing time opportunity was set in this study. If the participants viewed the time closer to the target, the remaining time interval was shorter. For example, in the limited viewing time condition, if some participants checked the time at 30 s, they would have to continue to estimate the remaining 30 s through internal effort. If he checked the time at 55 s, he only had to estimate the remaining 5 s. The deviation of individual estimated time is usually positively correlated with the length of the time interval (Smith et al., 2007). Therefore, smaller individual estimates of residual time intervals result in smaller deviation values. This is obviously conducive to the correct execution of TBPM tasks. Compared with internal attention, external attention is more cost‐effective (Guo et al., 2022). Therefore, how participants in the limited viewing time condition used the only opportunity to check the time was very important. In the unlimited viewing time condition, participants could either check the time frequently or improve the accuracy of time estimation by checking the time more effectively. Therefore, compared with the unlimited viewing time condition, the promotional effect of prosocial motivation on TBPM when viewing time was limited may depend more on improving the effectiveness of external attention.

In terms of strategies, we found that the prosocial motivation group used more strategies than the control group under both viewing time conditions. This indicates that prosocial motivation prompted individuals to adopt more strategies to ensure the successful execution of the TBPM task. However, we further classified the strategies adopted by the participants under different viewing time conditions and found that the strategies differed in the two time‐monitoring conditions. When viewing time was limited, strategic viewing time ranked first as the strategy adopted by the participants, that is, the participants alternated time estimation and viewing time to ensure the successful execution of TBPM. The second most used strategy was frequently checking the time. However, in the limited viewing time condition, the strategy reported by participants that ranked first in terms of quantity was making the best use of the only viewing time opportunity, that is, making more effort to ensure that the first‐time estimate was as accurate as possible, and then estimate the remaining time. The second most popular strategy was estimating the time by counting. In conclusion, participants mainly adopted strategies related to external attention under the unlimited viewing time condition, while participants chose the strategy of ensuring time estimation under the unlimited viewing time condition. Therefore, although the number of prosocial motivation strategies under the two time‐monitoring conditions was greater, the strategies they adopted in the different time‐monitoring conditions were different. Finally, we found no difference in strategy effectiveness between the prosocial motivation group and the control group, indicating that prosocial motivation did not prompt individuals to adopt more effective strategies. This may be because a considerable number of effective strategies depend upon the individual's own abilities. For example, more accurate estimation of a time interval requires individuals to have better time estimation abilities (Gan & Guo, 2019). Although individuals can better estimate time by counting, this method requires a high level of working memory ability (Camos & Barrouillet, 2004), so only individuals with high working memory abilities may be able to use counting as an effective strategy without seriously interfering with ongoing tasks. Although external strategies, such as reminders, are highly cost‐effective (Ryder et al., 2022), as this study was a typical laboratory study, individuals could not easily set reminders as they do in daily life. Therefore, when studying what types of strategies individuals adopt under prosocial motivation conditions to ensure the completion of prospective memory tasks, experimental scenarios with higher ecological validity should be used.

This study also found that the attention consumption and strategies used by the prosocial motivation group increased, which was consistent with the prediction of the motivational cognitive model. However, the motivational cognitive model does not specify what changes occur in internal and external attention and what strategies are adopted. Moreover, the motivational cognitive model does not explain how prosocial motivation promotes TBPM under different time‐monitoring conditions. This study focused on these aspects to supplement the motivational cognitive model. It is worth noting that the motivational cognitive model states that strategies can improve the automatic processing of TBPM tasks, which is an example of an automatic processing approach, while improving TBPM performance by paying more attention is an example of controlled processing. These two approaches are independent of each other (Penningroth & Scott, 2014). However, this study found that internal attention, external attention, and the use of strategies were closely related. For example, the strategy that individuals adopted the most under the unlimited viewing time condition was strategic viewing time, which requires the alternation of internal and external attention. In addition, in the limited viewing time condition, individuals must pay more internal attention to ensure that the first viewing time is more accurate, and the counting method also consumes additional internal attention. This is not a method of automatic processing as predicted by the motivational cognitive model, but a controlled processing approach.

The findings of this study extend the application scope of motivational cognitive model and have certain theoretical significance. However, this study has some limitations. First, the TBPM task interval set in this study was relatively short, and there are differences in the processing mechanisms of long‐duration TBPM and short‐duration TBPM. The estimation of short‐duration TBPM can be successfully completed by relying on continuous internal attention (Guo et al., 2019). However, it is unrealistic to continuously estimate the time when estimating long‐duration TBPM, in which case people rely more on external time information (Belmar et al., 2020). Therefore, the processing mechanism by which TBPM promotes prosocial motivation may also be different with different time intervals, and the results of this study may only be applicable to short‐duration TBPM tasks. Secondly, the TBPM task intended behavior adopted in this study was relatively simple, and participants only needed to remember simple reactions. Individuals could correctly perform TBPM tasks only through effective time processing. However, when the intended behavior is more difficult, individuals need to pay more attention to the retention and retrieval of intended content (Meier & Zimmermann, 2015). Therefore, the processing mechanism of TBPM with different intended behavior difficulty levels may be different. The results of this study can only be applied to TBPM tasks with easy intentional behavior, and cannot be blindly extended to other conditions.

## CONCLUSION

This study focused on how prosocial motivation promoted TBPM under two time‐monitoring conditions. The results revealed that prosocial motivation could increase individual TBPM performance under both limited and unlimited viewing time conditions, with different mechanisms underpinning the social motivation promotion of TBPM in each condition. When viewing time was unlimited, prosocial motivation prompted individuals to spend more internal and external attention and improved the effectiveness of external attention. When viewing time was limited, however, prosocial motivation prompted individuals to improve internal attention consumption and external attention effectiveness. In addition, prosocial motivation prompted individuals to adopt different strategies in the different time‐monitoring conditions. When viewing time was unlimited, individuals mainly adopted strategies related to time‐monitoring, and by comparison, they mainly adopted strategies to ensure the accuracy of time estimation when viewing time was limited.

## CONFLICT OF INTEREST STATEMENT

The authors declare there are no conflicts of interest.

## ETHICS STATEMENT

This study was reviewed and approved by Institutional Review Board of Henan Provincial Key Laboratory of Psychology and Behavior (reference: 20220715001).
